# Dataset of the microbiome composition in skin lesions caused by lumpy skin disease virus *via* 16s rRNA massive parallel sequencing

**DOI:** 10.1016/j.dib.2019.104764

**Published:** 2019-11-06

**Authors:** Sören Hansen, Rodrigo Pessôa, Andrezza Nascimento, Mohamed El-Tholoth, Ahmed Abd El Wahed, Sabri S. Sanabani

**Affiliations:** a Division of Microbiology and Animal Hygiene, University of Goettingen, Burckhardtweg 2, D-37077, Göttingen, Germany; b Laboratory of Dermatology and Immunodeficiencies, LIM-56, Department of Dermatology, Tropical Medicine Institute of São Paulo, University of São Paulo, Av. Dr. Enéas Carvalho de Aguiar, 470 - Jardim America, São Paulo, SP, 05403-000, Brazil; c Department of Virology, Faculty of Veterinary Medicine, Mansoura University, 35516, Mansoura, Egypt

**Keywords:** Lumpy skin disease virus, Microbiome, 16s rRNA sequencing

## Abstract

Lumpy Skin Disease (LSD) is a highly contagious viral disease affecting cattle mainly and induced by the Lumpy Skin Virus within the Capripoxvirus genus of the family Poxviridae. LSD infected animals exhibit pyrexia and sudden appearance of localized or generalized skin nodules that may slough leaving ulcers. The disease has negative economic impacts as a result of hide damage, mastitis, infertility and losses in milk production. Secondary bacterial infection in the affected skin lesions can increase the severity and prolong the course of the disease. Little is known about the microbiome in the ulcerated skin sites. Therefore, the present study was directed to identify the prevalent bacterial communities in affected lesion *via* the 16s rRNA gene sequencing. Up to 98 species were found in the samples, most of them belonging to the phyla of Proteobacteria, followed by Firmicutes, Actinobacteria, and Bacteroidetes. All found bacterial species are known as opportunistic pathogens, but can withstand the inflammatory reaction.

**Table:** Specifications

Subject	Veterinary Science
Specific subject area	Determination of the bacterial composition of the microbiome in the lesions of a Lumpy Skin disease virus (LSDV) infected cattle.
Type of data	Table Figure Dataset of bacterial species
How data were acquired	16s rRNA massive parallel sequencing of DNA extracted from skin biopsy Instruments: MiSeq Sequencer (Illumina, San Diego, CA, USA), PANDAseq v.2.9 software [1], UCHIME algorithm [2], EzTaxon-e database [3], Mothur [4], and Shannon-ace-table.pl software programs (Chunlab Inc., Seoul, Korea)
Data format	Raw Analyzed
Parameters for data collection	Data were collected from biopsy samples of RPA confirmed LSDV positive animals. Samples were taken after oral consent was given by the owner following the national ethical regulations.
Description of data collection	Six samples of LSDV-affected skin were biopsied under sterile conditions from each animal. The samples were collected after regular cattle slaughtering at abattoir.
Data source location	City/Town/Region: Dakahlia Governorate Country: Egypt
Data accessibility	Repository name: ZENODO Data identification number: [1256899] Direct URL to data: https://zenodo.org/record/1256899#.XVupcq35y9Y

**Table:** 

**Value of the Data** • The data show the change of the bacterial diversity, due to the different species habitat during inflammation. Although all bacteria found are environment associated, they may overwhelm the immune system and become pathogenic leading to more severe conditions. • The data can be used to aid the veterinarians in the selection of the right treatment and the virologist to study the LSDV pathogenesis. • The dataset paves the way for a better insight into the course of the Lumpy Skin Disease, especially the secondary bacterial infection. Experiments can use the dataset as a base of further investigations of the disease and the immunological reactions it triggers in the animal, beside the development of a disease progression marker • The dataset provides an extraordinary insight into the links between environment associated bacteria and inflammatory lesions as a cause of viral infections. This can help to evaluate the pathogenic potential of these bacteria towards the diseased subject.

## 1 Data

**Fig. 1:**
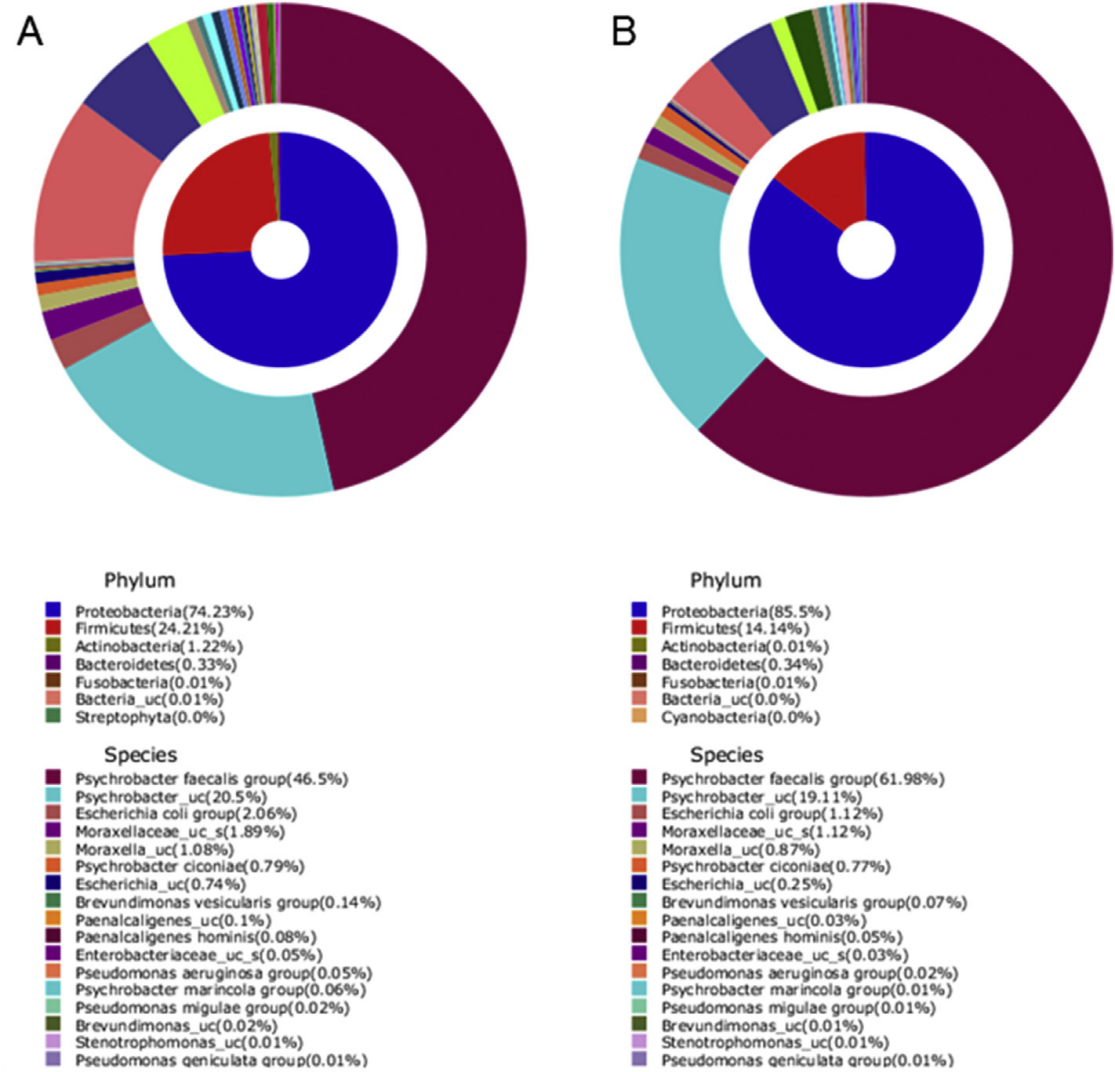
Average composition of bacteria in pool sample group 1 (A) and 2 (B).

The dataset contains the microbiome analysis of pooled DNA samples isolated from six skin biopsies of lumpy skin disease virus infected cattle. The raw data files were deposited in ZENODO.ORG under reference number: 1256899. The composition of the bacteria in the infected tissues was illustrated in Fig. 1 and Table 1.

**Table 1:** Classification of the most detected bacterial subspecies.

Sample			Most detected Phylum	No. of reads identified	Most detected Families	No. of reads identified	Most detected Subspecies	No. of reads identified	Percentage
LSDV_N01			*Proteobacteria*	74,081	*Moraxellaceae*	70,690	*Psychrobacter faecalis* group	46,403	49,49
							*Psychrobacter_uc*	20,453	21,81
					*Enterobacteriaceae*	*2841*	*Escherichia coli* group	2058	2,19
					*Caulobacteraceae*	156	*Brevundimonas vescularis* group	136	0,15
					*Pseudomonadaceae*	89	*Pseudomonas aeruginosa* group	48	0,05
			*Firmicutes*	24,155	*Clostridiaceae*	14,431	*Clostridium tertium* group	10,919	11,65
							*Clostridium_uc*	2823	3,01
							*Clostridium senegalense*	569	0,61
					*Peptostreptococcaceae*	6400	*Clostridium mangenotii*	5760	6,14
							*Clostridium_g4_uc*	634	0,68
					*Bacillaceae*	1359	*Bacillus cereus* group	602	0,64
							*Bacillus_uc,* DQ345456_s	227	0,24
							HM839572_s	29	0,03
					*Erysipelotrichaceae*	1190	*Erysipelothrix*	435	0,46
							*Erysipelotrichaceae_uc*	755	0,81
					*Planococcaceae*	548	*Sporosarcina_uc*	139	0,15
							*Sporosarcina koreensis* group	129	0,14
							*Sporosarcina urea* group	49	0,05
					*Enterococcaceae*	522	*Vagococcus fluvialis*	291	0,31
							*Vagococcus _uc*	87	0,09
							*Enterococcus fecalis*	54	0,06
							*Enterococcus casselilavus* group	40	0,04
			*Actinobacteria*	1220	*Micrococcaceae*	1141	*Glutamicibacter creatinolyticus*	681	0,73
							*Glutamicibacter_uc*	439	0,47
LSDV_N02			*Proteobacteria*	85,326	*Moraxellaceae*	*83,696*	*Psychrobacter faecalis* group	61,851	64,51
							*Psychrobacter_uc*	19,067	19,89
					*Enterobacteriaceae*	1390	*Escherichia coli* group	1114	1,16
			*Firmicutes*	14,107	*Peptostreptococcaceae*	5039	*Clostridium mangenotii*	4625	4,82
							*Clostridium_g4_uc*	409	0,43
			*Clostridiaceae*			4585	*Clostridium tertium* group	3508	3,66
							*Clostridium_uc*	988	1,03
							*Clostridium senegalense*	50	0,05
			*Enterococcaceae*			2352	*Vagococcus lutrae*	1788	1,86
							*Vagococcus _uc*	356	0,37
							*Vagococcus fluvialis*	151	0,16
			*Planococcaceae*			854	*HQ603002_s*	550	0,57
							*Savagea_uc*	219	0,23
			*Erysipelotrichaceae*			811	*Erysipelothrix_us*	567	0,59
			*Bacillaceae*			443	*Bacillus cereus* group	239	0,25
							*Bacillus_uc*	119	0,12
							*DQ345456_s*	59	0,06
			*Bacteroidetes*	341	*Bacteroidaceae*	218	*Bacteroides pyogenes*	164	0,17
							*Bacteroides_uc*	54	0,06

“_uc” stays for unclassified. This may indicate that reads have insufficient signal in the sequenced region to allow their classification on subspecies level or they are novel species.

## 2 Experimental design, materials, and methods

### 2.1 Sampling and ethical statement

Six samples of LSDV-affected skin were biopsied under sterile conditions from slaughtered cattle (n = 2) Egypt. The samples were collected after regular cattle slaughtering at the abattoir in Dakahlia Governorate, Egypt. Oral consent was given by the owner following the national ethical regulations. The biopsies were maintained at −80 °C without formalin until testing. The presence of the LSDV in the collected samples was confirmed by real-time RPA as previously described [5].

### 2.2 DNA extraction

*The PowerSoil DNA kit (MO BIO Laboratories™: Carlsbad, CA, USA) was applied to extract the DNA.* Briefly, eight-millimeter of skin biopsies from infected tissue were ground with a mortar and pestle under sterile condition. The digested tissues were then added to the Powerbead tubes contained ceramic beads and 60 μl lysis buffer. The contents of each tube were mixed by vortexing at maximum speed for 10 minutes. Thereafter, 60 μL of solution C1 were added and the tubes were gently vortexed for 5 seconds. The tubes were centrifuged at 10,000×*g* at room temperature for 30 sec and the supernatants were transferred to clean tubes. The supernatant was mixed with 250 μl of Solution C2 and vortexed for 5 seconds. Consequently, the tubes were incubated at 4 °C for 5 min and then centrifuged at 10,000×*g* for one min. The supernatant was transferred to a separate clean collection tube. For further removal of inhibitor, 200 μl of the non-DNA organic and inorganic material removal solution (Solution C3) were added to the supernatant and incubated at 4 °C for 5 min. Following that, the tubes were centrifuged at 10,000×*g* for one min and the supernatants were transferred to a 2 ml tube. Then, 1.2 ml of high concentration salt solution (Solution C4) was added to the supernatant and the mixtures were quickly vortexed. The mixtures were loaded onto a spin filter and centrifuged at 10,000×*g* at room temperature for 1 min. Five hundred microliters of ethanol-based washing solution (Solution C5) were added and centrifuged at 10,000×*g* at room temperature for 30 sec. The spin filters were centrifuged again at 10,000×*g* for one min to get rid of all traces of ethanol. One hundred microliter of elution buffer (Solution C6) were added and centrifuged at 10,000×*g* for 30 seconds at room temperature. The DNA in the flow through were used for library preparation and sequencing.

### 2.3 Sample preparation and sequencing

DNA from each sample was pooled at equal concentration. In triplicates, the V4 region of the 16S rRNA gene was amplified using the Bakt_341F (5′-CCTACGGGNGGCWGCAG-3′) and Bakt_805R (5′-GACTACHVGGGTATCTAATCC-3′) [6]. Two amplification cycles were performed using the Illumina barcode and adaptors as well as the Phusion Hot start II polymerase (Thermo Fisher Scientific Inc., Waltham, Massachusetts, United States). In the first amplification, twenty-two cycles were conducted with annealing temperature at 50 °C to amplify the 16S gene and add the barcode as well as partial Illumina adaptor. In the second amplification, 12 cycles were deployed to assure the attachment of the remaining ends of the Illumina adaptors, for the detailed protocol please refer to Refs. the published protocol [7,8]. The product was run onto gel and the 464 bp amplicons were extracted and purified employing the Freeze N Squeeze DNA Gel Extraction Spin Columns (Bio-Rad: Hercules, CA, USA). The DNA content was measured by a Qubit 2.0 Fluorometer (Life Technologies: Carlsbad, CA, USA) and an equimolar contraction of each amplicons was diluted to 4 nM. The steps of DNA indexing and library preparations were conducted as previously reported [8]. Briefly, The DNA was denaturated by incubation at room temperature for 5 minutes with 0.2 N fresh NaOH. Thereafter, 990 μl Illumina HT1 buffer were added to the mix. To increase sequence diversity, 20 pM library was multiplexed with 6 μL of 12.5 pM denatured PhiX control. An of 234 μL of chilled HT1 buffer was added to make a 12 pM library. The pooled libraries were loaded into an Illumina MiSeq cartridge for paired end 300 sequencing.

Initially, image analysis, base calling, and data quality assessment took place on the MiSeq instrument (San Diego, CA, USA). The PANDAseq v.2.9 software [1] was used to assemble the paired-end reads into single sequence. The potential recombinant sequences were omitted by the UCHIME algorithm [2]. The EzTaxon-e database [3] was applied to classify bacterial strain with a threshold of 97% pairwise sequence identity. Mothur [4] and Shannon-ace-table.pl software programs (Chunlab Inc., Seoul, Korea) were utilized to compute the bacterial community richness indices (non-parametric Chao1) and diversity indices (Shannon estimator).
